# Identification and validation of miR-583 and mir-877-5p as biomarkers in patients with breast cancer: an integrated experimental and bioinformatics research

**DOI:** 10.1186/s13104-023-06343-w

**Published:** 2023-05-08

**Authors:** Zahra Foruzandeh, Mohammad Reza Alivand, Mehdi Ghiami-Rad, Mohammad Zaefizadeh, Saeid Ghorbian

**Affiliations:** 1 Department of Molecular Genetics, Ahar Branch, Islamic Azad University, Ahar, Iran; 2 Eye Research Center, The Five Senses Health Institute, Rassoul Akram Hospital, Iran University of Medical Sciences, Tehran, Iran; 3 Stem Cell and Regenerative Medicine Research Center, Iran University of Medical Sciences, Tehran, Iran; 4 Department of Microbiology, Faculty of Basic Sciences, Ahar Branch, Islamic Azad University, Ahar, Iran; 5 Ardabil Branch, Islamic Azad University, Ardabil, Iran

**Keywords:** MicroRNA, miR-583, Biomarker, Breast cancer, miR-877-5p

## Abstract

**Objectives:**

Breast cancer (BC) is one of the most common cancers with a high mortality rate in women worldwide. The advantages of early cancer diagnosis are apparent, and it is a critical factor in increasing the patient’s life and survival. According to mounting evidence, microRNAs (miRNAs) may be crucial regulators of critical biological processes. miRNA dysregulation has been linked to the beginning and progression of various human malignancies, including BC, and can operate as tumor suppressors or oncomiRs. This study aimed to identify novel miRNA biomarkers in BC tissues and non-tumor adjacent tissues of patients with BC. Microarray datasets GSE15852 and GSE42568 for differentially expressed genes (DEGs) and GSE45666, GSE57897, and GSE40525 for differentially expressed miRNAs (DEMs) retrieved from the Gene Expression Omnibus (GEO) database were analyzed using “R” software. A protein-protein interaction (PPI) network was created to identify the hub genes. MirNet, miRTarBase, and MirPathDB databases were used to predict DEMs targeted genes. Functional enrichment analysis was used to demonstrate the topmost classifications of molecular pathways. The prognostic capability of selected DEMs was evaluated through a Kaplan-Meier plot. Moreover, the specificity and sensitivity of detected miRNAs to discriminate BC from adjacent controls were assessed by area under the curve (AUC) using the ROC curve analysis. In the last phase of this study, gene expression on 100 BC tissues and 100 healthy adjacent tissues were analyzed and calculated by using the Real-Time PCR method.

**Results:**

This study declared that miR-583 and miR-877-5p were downregulated in tumor samples in comparison to adjacent non-tumor samples (|logFC|< 0 and P ≤ 0.05). Accordingly, ROC curve analysis demonstrated the biomarker potential of miR-877-5p (AUC = 0.63) and miR-583 (AUC = 0.69). Our results showed that has-miR-583 and has-miR-877-5p could be potential biomarkers in BC.

## Introduction

With an estimated 1,200,000 new cases each year, Breast cancer (BC) is the most frequently diagnosed malignancy in females [1, 2]. BC is a complex and heterogeneous disease that can be classified into several subtypes based on histopathological characteristics, tumor grade, and lymphovascular invasion [3]. However, it sometimes uncovers after signs emerge, despite many women with BC having no symptoms [4], and neither of the commonly used methods is accurate enough to meet the criteria for diagnosing BC, and the late diagnosis is one of the reasons for the high mortality rate from this cancer. The authors list surgery, chemotherapy, and radiation as main treatment plans for patient with breast cancer. However, since majority of newly-diagnosed breast cancers are hormone-receptor positive and the patients receive endocrine therapies, targeted therapies should be listed as a treatment plan [5]. Despite breakthroughs in treatment, approximately half of people with BC will have a cancer recurrence or die within 5 years [6]. Therefore, new biomarkers associated with gene expression must be found to assist valuable diagnostic procedures and treatment strategies for BC patients by increasing the analysis of molecular pathways linked to the onset and progression of the tumor [7–10].MicroRNAs (miRNAs) are small single-stranded non-coding RNAs that hinder the expression of their target genes [11, 12], are crucial regulators for essential cellular functions, and have a role in different disorders [13] and various cancers [14, 15]. MiRNAs are involved in tumorigenesis via altering the expression of tumor suppressor genes and oncogenes [16], and they are also important for the cell cycle [17], migration and invasion [18], apoptosis [19], metastasis [20], and the development of cancer [21]. Although miRNA expression patterns are consistent across all cancer types, most of them are cell-type-specific and might thus be used as cancer biomarkers. Evidence from malignant tissues and cell lines shows that miRNAs have a role in the development of all types of BC [22–24]. Evidence suggests that miRNAs, a form of small endogenous singlestranded RNA that binds to the 3’ untranslated region of purpose mRNAs, may play essential roles in numerous biological processes and are proven to negatively suppress gene expression. However, numerous cellular processes are impacted by miRNAs, containing proliferation, differentiation, apoptosis, migration, metabolism, and stress response [25]. High-throughput genomic technologies have greatly helped researchers in understanding the mechanisms involved in cancer pathogenesis by allowing them to explore the involvement of genetic variables in cancer. Furthermore, the development of novel methods, such as enrichment analysis and prediction tools, enables more extensive investigations of molecular networks and disease pathophysiology processes and provides an exceptional tool for identifying novel therapeutic targets. Seizing the benefit of microarray datasets and other in situ tools, we aimed to find novel markers for earlier and more accurate identification to evaluate the expression levels and diagnostic values of miRNAs and validate our data through experimental analyses between BC tissues and non-tumor adjacent samples to find novel markers for earlier and more accurate identification.

## Materials and methods

### Microarray-based mRNA and miRNA dataset selection

**Fig. 1:**
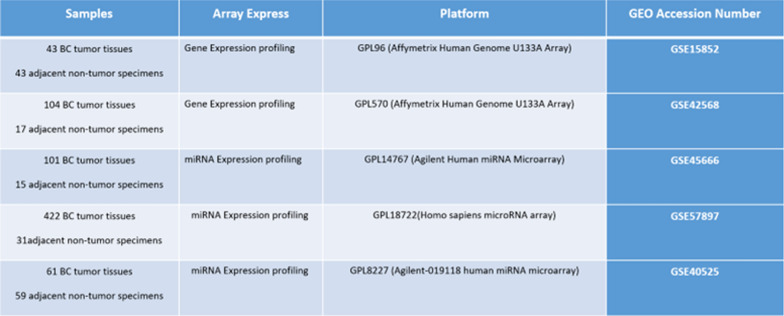
GEO Datasets information

By searching for keywords “Breast Cancer and adjacent normal controls”, GSE15852 and GSE42568 mRNA datasets, and GSE45666, GSE57897, and GSE40525 miRNA datasets were screened and downloaded from the National Center for Biotechnology Information (NCBI) Gene Expression Omnibus (GEO) database (http://www.ncbi.nlm.nih.gov/geo/). The information of the datasets is available in (Fig. 1).

### Identification of DEMs and DEGs

The “R” software and “LIMMA” package were employed to efficiently analyze the chosen datasets utilizing correction, quantile normalization, and log2 conversion. The screening criteria were |log2 fold-change (FC)| ≤ − 0.5 and FDR adjusted p ≤ 0.05 for DEMs, and |log2 fold-change (FC)| ≥ 1 and FDR adjusted p-value ≤ 0.05 for DEGs [26].

### Pathway enrichment and functional analysis

Then, DEGs were investigated utilizing the Kyoto Encyclopedia of Genes and Genomes (KEGG) pathway enrichment via the EnrichR database. The p < 0.05 was described as a significant enrichment analysis outcome. Potential roles were anticipated using KEGG pathway analysis.

### Validation of the hub miRNAs

We aimed to explain a certain discrimination capacity of these miRNAs for cancer and non-cancer tissues and the diagnostic power of the hub miRNAs by plotting the receiver operating characteristic (ROC) curve and estimation of the area under the curve (AUC).

### Ethics statement

All methods were performed in accordance with the guidelines of the Helsinki and was approved by the Research Ethics Committee of Azad University of Medical Sciences, Tabriz, Iran with Ethics code IR.IAU.TABRIZ.REC.1401.103. The patients’ written informed permission was gathered before participation.

### Study Population

Fresh-frozen specimens from 200 breast tumors were obtained from patients with BC undergoing surgery at Tabriz’s Al-Zahra Hospital from 2020 to 2022. After that, a histological examination was used to investigate the invasion and spread of cancer cells. As controls, tumor margin samples that a pathologist deemed to be healthy were also collected from a region of the resected specimen at the farthest distance from the tumor. The specimens were stored at − 80 °C instantly. None of the patients had been treated with preoperative radiotherapy, chemotherapy, or other relevant conditions. All patients signed informed permissions and agreed to use their surgical samples for investigation. The pathological characteristics of patients are shown in (Table 1).

**Table 1:** Primary demographic features of patients with BC

Parameter	Number of patients (Sample size)		Percentages (%)
Age	≤ 50	103	51.5
	> 50	97	48.5
Abortion history	Yes	101	50.5
	No	99	45.5
Family cancer history	Yes	75	60
	No	49	39.5
Tumor size	> 10 cm^3^	73	54
	> 10 cm^3^	49	46

### RNA extraction and cDNA synthesis

After homogenizing the tissues with liquid nitrogen, TRIzol reagent (Geneall) was added to extract the total RNA content from tissues. After that, a NanoDrop spectrometer (Thermo Scientific, USA) was utilized to evaluate the amount and quality of the isolated RNAs. Following extraction, the RNAs were purified in 50 µL of RNase-free water and kept at − 80 °C for storage. Reverse transcriptase (RT) enzyme (Thermo Fisher, USA), dNTP (Cinnaclone, Iran), and the specific stem-loop primers were used to create cDNA for miR-583, miR-877-5p, and RNU6 (for normalization). Three distinct stem-loop primers were created for miR-583, miR-877-5p, and RNU6, specially designed for this purpose. The reaction requirements were to hold step at 95 °C for 10 min, followed by 40 cycles of denaturation at 94 °C for 15 s, annealing at 62 °C for 30 s, and extension at 72 °C for 20 s. The final product was kept at 4 °C for preservation. The sequences of the primers are demonstrated in (Table 2**)**.

**Table 2:** Primer sequences for cDNA synthesis and real‑time PCR.

	MiRNAs and their accession numbers	Sequences	
cDNA Synthesis reaction	hsa-mir-887-5p (MIMAT0004949)	hsa-miR-877-5p(STL)	5ʹGTCGTATCCAGTGCAGGGTCCGAGGTATTCGCACTGGATACGACCCCTGC3ʹ
	hsa-mir-583 (MIMAT0003248)	hsa-mir-583(STL)	5ʹGTCGTATCCAGTGCAGGGTCCGAGGTATTCGCACTGGATACGACGTAATGG3ʹ
	RNU6 NR_003027.2	U6(STL)	5′GTCGTATCCAGTGCAGGGTCCGAGGTATTCGCACTGGATACGACAAAAATAT3ʹ
Real time PCR reaction	hsa-mir-887-5p (MIMAT0004949)	hsa-miR-877-5p(F)	5′GTAGAGGAGATGGCGCAGGG3′
	hsa-mir-583 (MIMAT0003248)	hsa-mir-583(F)	5′CCCAAAGAGGAAGGTCCCATTAC3′
	RNU6 NR_003027.2	U6(F) U6(R)	5′GCTTCGGCAGCACATATACTAAAAT3′ 5′CGCTTCACGAATTTGCGTGTCAT3′
	hsa-mir-877-5p and hsa-mir-583	Common(R)	5′GTGCAGGGTCCGAGGT3′

### Data analysis

The target DEMs ratio between the BC tissues and non-tumor adjacent tissues was indicated by the 2− ^(ΔΔCT)^, and the formula was as follows: R = 2^− (ΔΔCT)^.

$$\triangle\triangle{\text{CT}} = ({\text{CT target}} - {\text{CT reference}}) \, \text{healthy} - ({\text{CT target}} {\text{CT reference}}) \,\text{patient}$$

Threshold Cycle (CT) identified the amplification cycle when the reaction’s real-time fluorescence intensity approximated the determined threshold, and the amplification was in the logarithmic phase. Per experiment was conducted two times to define the mean value. Logarithm 2 of Fold Change (was used in simple linear regression) and Two- sample T-test to compare two groups of data were applied to compare the expression level of selected miRNAs between demographic features of the subgroups like age, family history, abortion history, and tumor size. Moreover, survival analysis was evaluated by the Kaplan-Meier survival plot designed by GraphPad prism 8.4.3 to assess the prognostic value of selected DEMs in BC patients. All results are presented as P-value < 0.05 and mean ± SEM using the GraphPad Prism 8.4.3 and “R” software version 4.3.1.

## Results

### Identification of DEMs and DEGs

**Fig. 2:**
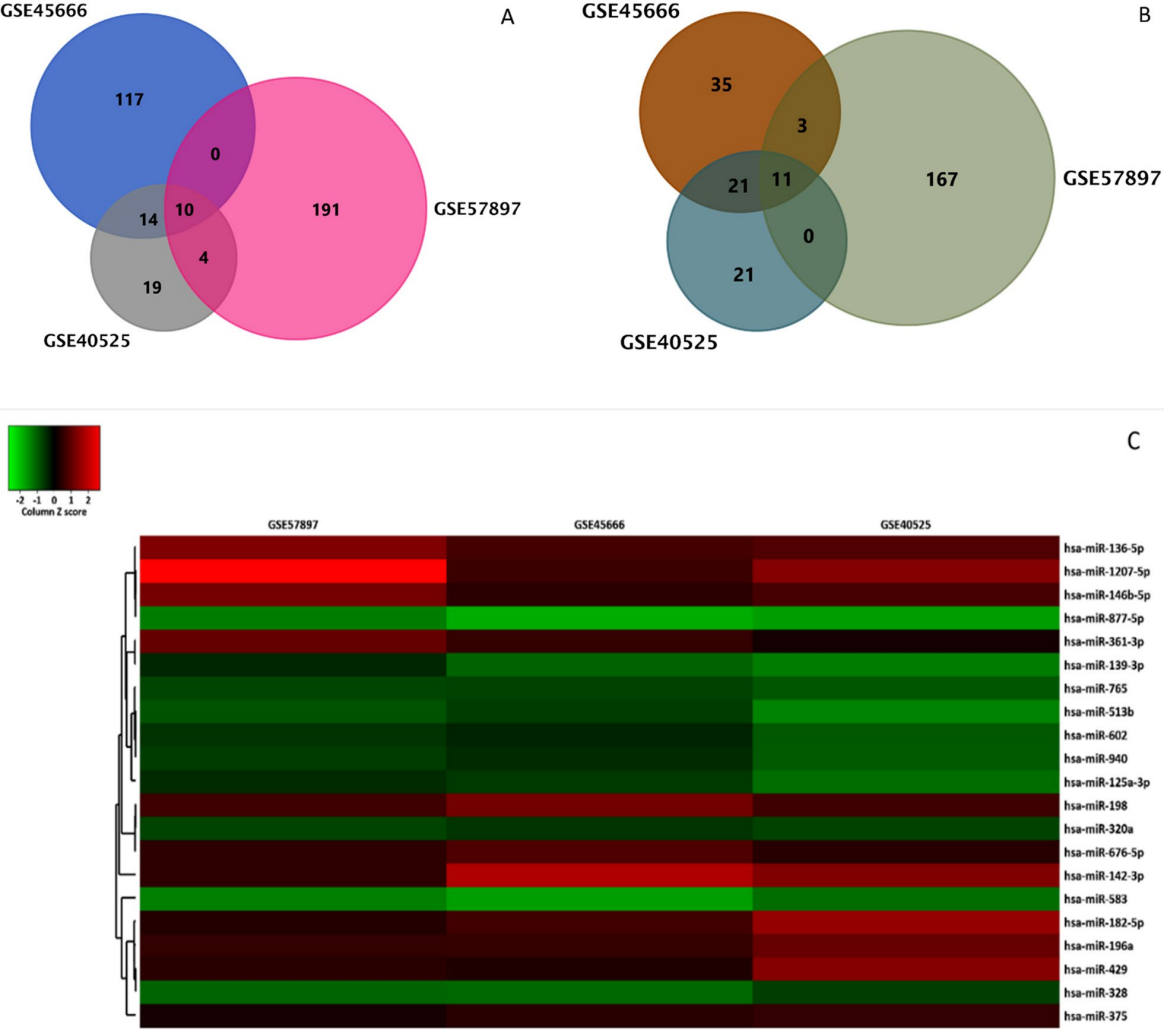
DEMs in expression array of GSE57897, GSE4566, and GSE40525 datasets. **A** Venn diagram of common downregulated DEMs. **B** Venn diagram of common upregulated DEMs. **C** log2FC heatmap of DEMs in GSE57897, GSE4566, and GSE40525. Red rows are upregulated while green rows are downregulated

**Fig. 3:**
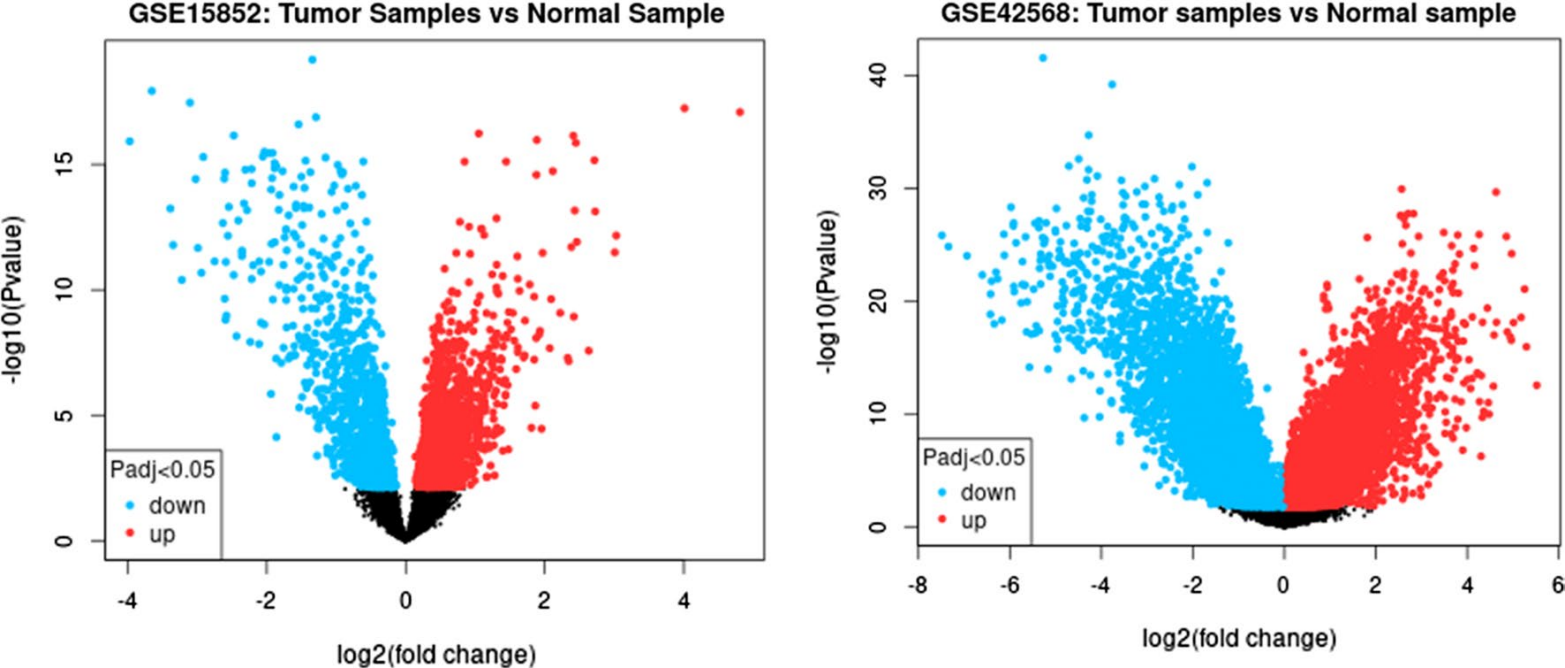
Differential volcano plots of DEGs in BC tissues versus adjacent normal tissues in GSE15852 and GSE42568 databases

Considering |logFC| ≤ − 0.5 and p < 0.05 criteria for DEMs, 386 DEMs (181 upregulated and 205 downregulated) were identified in GSE57897, 213 DEMs (71 upregulated and 142 downregulated) in GSE45666, and 105 DEMs (54 upregulated and 51 downregulated) in GSE40525 **(**Fig. 2A and B, and 2 C**)**. Furthermore, |logFC| ≥ 1 and p < 0.05 criteria for differentially expressed genes resulted in 410 DEGs (177 upregulated and 158 downregulated) in GSE15582 and 233 DEGs (25 upregulated and 208 downregulated) in GSE42568. Differentially expressed plot of each dataset is presented in **(**Fig. 3**)**.

### Detection of mRNA-miRNA network

**Fig. 4:**
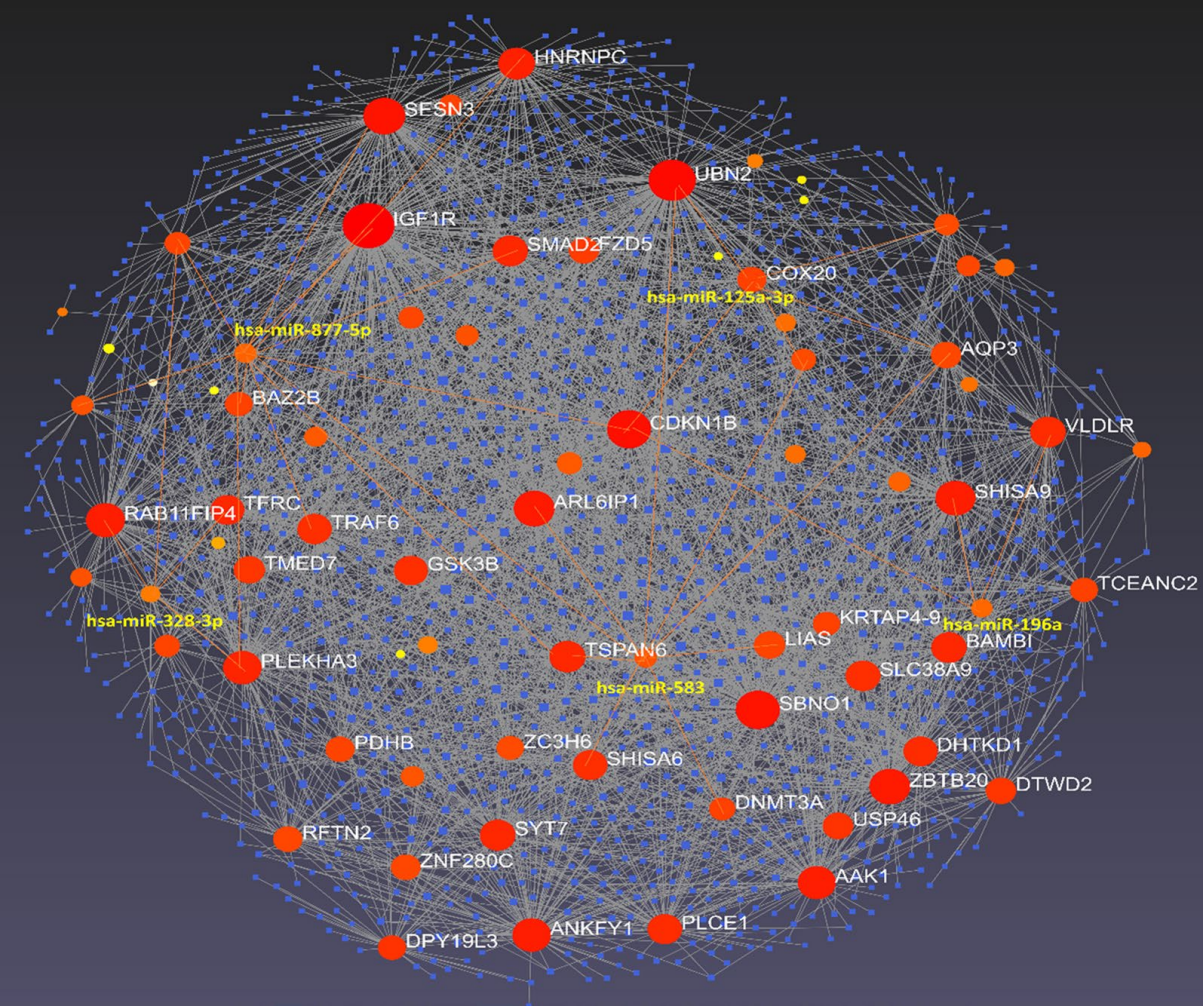
PPI network from minimum network and the selected module. Red nodes with labels indicated DEGs, and orange nodes with yellow labels show hub DEMs.

The establishment of a miRNA-mRNA network was carried out using the detected DEMs and DEGs by miRNet [27] and String database [28] **(**Fig. 4**)**. As expected, hsa-miR-583 and hsa-miR-877-5p have a direct influence on the greatest number of DEGs and are regarded as hub miRNAs.

### Screening enrichment analysis of common DEGs

Each of the target genes analyzed by microarray datasets has their pathways enriched. KEGG pathway analysis [29]demonstrated that the potential target genes were mainly enriched in 10 pathways presented in **(**Table 3**)**.

**Table 3:** KEGG Enrichment Analysis of miR-583 and miR-877-5p target genes

KEGG ID	Pathway	Genes	P.value	FDR
hsa04144	Endocytosis	SMAD2, TFRC, CLTC, CBL, IGF1R, PLD2, CDC42, TRAF6, MVB12B, STAMBP, EPS15, VPS36, RAB11FIP4	3.61E-10	5.3E–l8
hsa05200	Pathways in cancer	CDC42, SMAD2, GSK3B, CDKN1B, FZD5, TRAF6, KIT, CBL, SOS2, IGF1R, PLD2	6.83E-05	5.1E–3
hsa05205	Proteoglycans in cancer	CDC42, SMAD2, FZD5, PLCE1, CBL, SOS2, IGF1R	2.43E-04	4.1E–9
hsa04014	Ras signaling pathway	CDC42, KIT, PLCE1, SOS2, IGF1R, PLD2	0.003458371	4.1E–5
hsa05224	Breast cancer	GSK3B, FZD5, KIT, SOS2, IGF1R	0.00381176	4.1E–1
hsa05226	Gastric cancer	SMAD2, GSK3B, CDKN1B, FZD5, SOS2	0.004000612	3.1E–8
hsa04150	mTOR signaling pathway	GSK3B, FZD5, SLC38A9, SOS2, IGF1R	0.004711629	2.1E–9
hsa04012	ErbB signaling pathway	GSK3B, CDKN1B, CBL, SOS2	0.006087197	2.1E–2
hsa05225	Hepatocellular carcinoma	SMAD2, GSK3B, FZD5, SOS2, IGF1R	0.006121151	1.1E–5
hsa05215	Prostate cancer	GSK3B, CDKN1B, SOS2, IGF1R	0.008764397	0.00084

### Validation of the hub miRNAs by the ROC curve

**Fig. 5:**
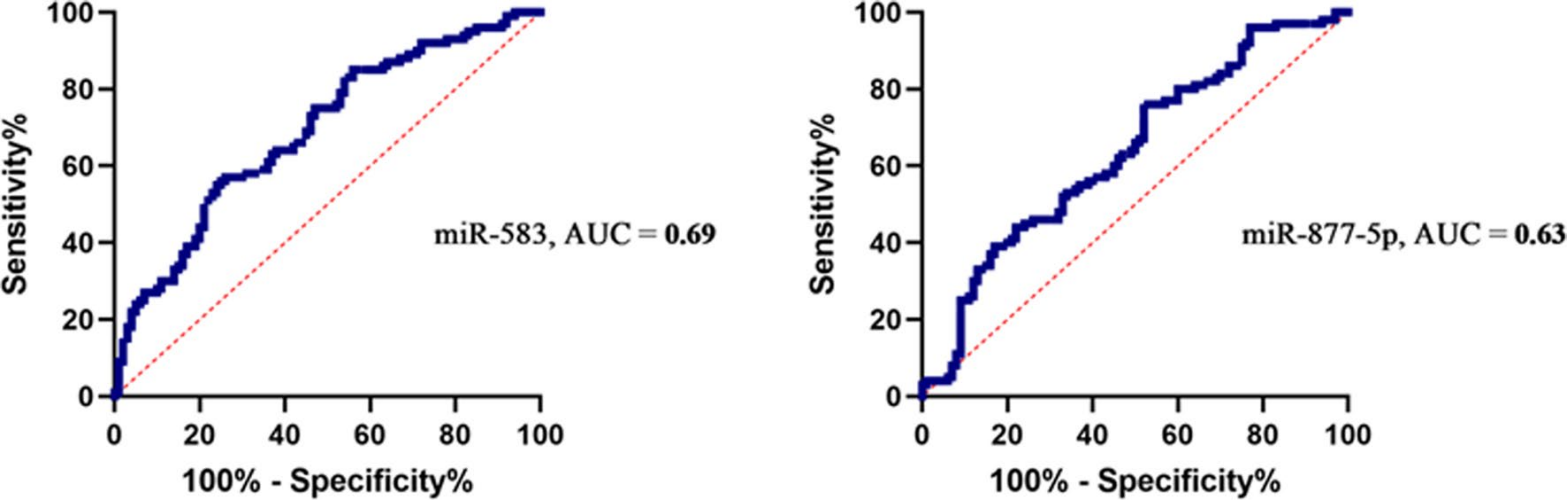
ROC curve analysis of miR-583 and miR-877-5p. Receiver operating characteristic (ROC) curve and area under the curve (AUC) statistics are used to evaluate the capacity to differentiate BC from healthy controls with good specificity and sensitivity

A ROC curve proved the diagnostic effectiveness of core miRNAs. The AUC indicated that miRNAs showed remarkable diagnostic efficiency for BC tissues and adjacent non-tumor tissues. Based on our results, miR-583 with AUC = 0.69 (specificity; 96.3%, sensitivity; 5.49%) and miR-877-5p with AUC = 0.63 (specificity; 96.3%, sensitivity; 5.49%) are good candidates for diagnosis of BC **(**Fig. 5**)**.

### Survival rate and prognostic value of the core DEMs

**Fig. 6:**
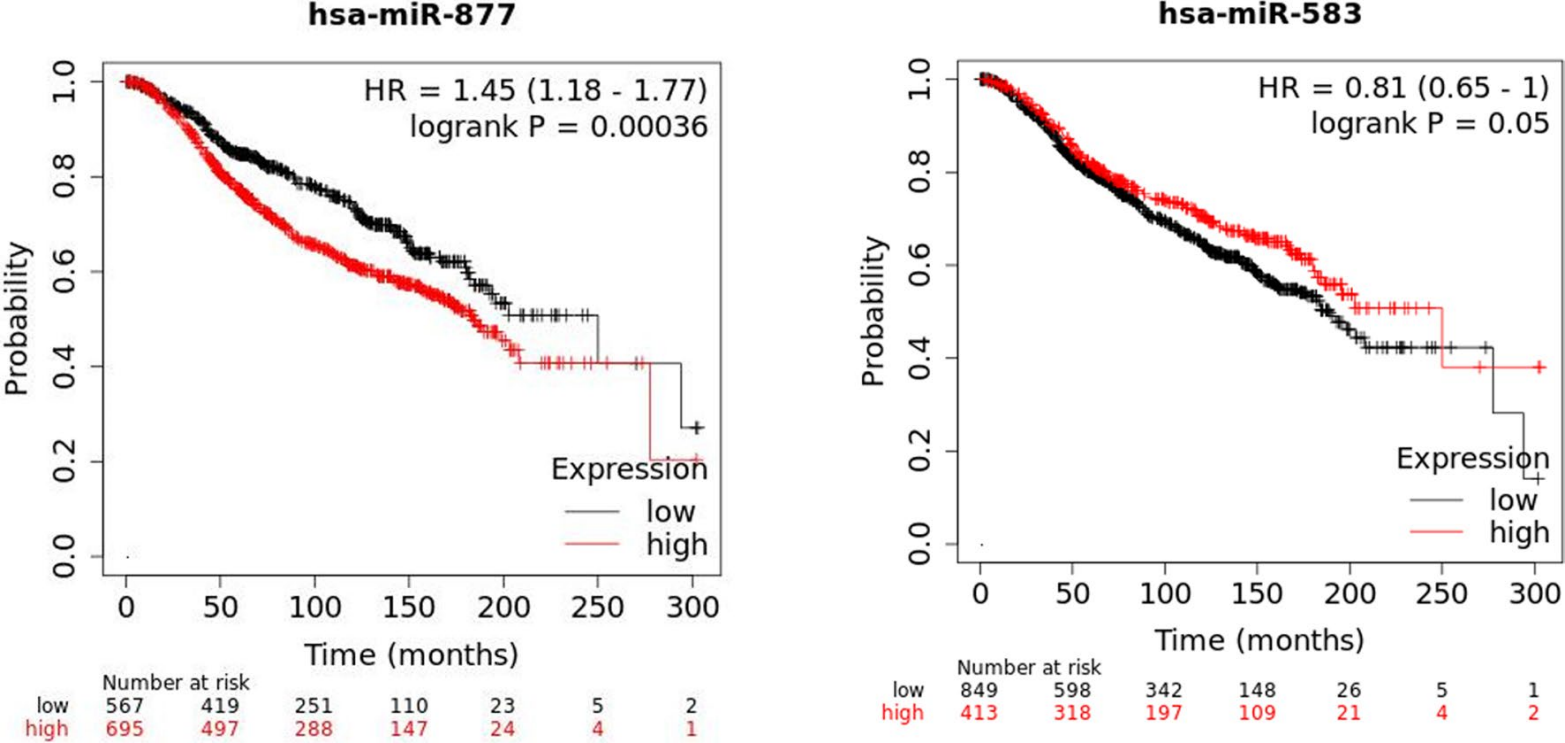
Two key miRNAs’ prognostic outcomes (Kaplan-Meier plots) in BC were examined using the METABRIC dataset

The METABRIC raw data, which possess an allied study of gene expression in the finding and confirmation of 1262 primary BC cases with ongoing clinical monitoring, were used to plot the Kaplan-Meier. The prognostic value of miR-583 and miR-877-5p was demonstrated in **(**Fig. 6**)**. The outcomes showed significant values for both miRNAs.

### Validation analysis using real- time PCR

**Fig. 7:**
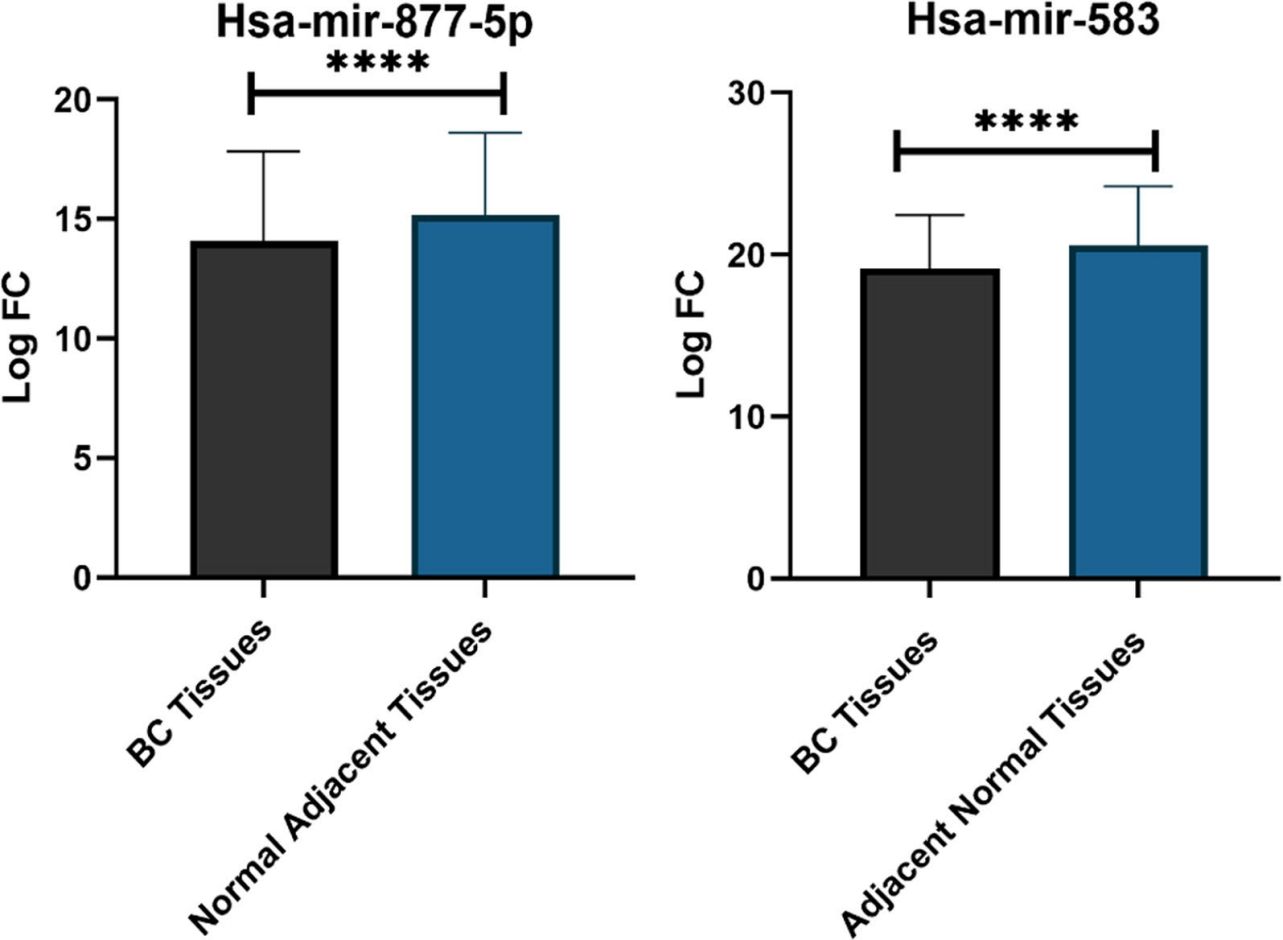
qPCR analysis for miRNA expression of BC samples and Adjacent non-tumor tissues. Diagram demonstrates fold change in expression of hub miRNAs. ****P < 0.0001

To understand much more about the effects of miR-583 and miR-877-5p in patients with BC, the expression of these miRNAs in BC samples and their non-tumor adjacent control samples was examined. By using real-time PCR, the expression of the chosen miRNAs was assessed in 200 BC tissues and matched with that of non-tumor adjacent samples **(**Fig. 7**)**. The expression of both hub miRNAs was aberrant and downregulated. The paired T-test findings revealed that the expression levels of miR-583 and miR-877-5p were considerably lower in human BC tissues than in non-tumor adjacent tissues, with FC = − 1.35 and FC = − 1.014, respectively.

### Subgroups analysis

LFC and Two-sample T-tests respectively were applied to analyze the correlation between the expression level of miR-583 and miR-877-5p with age, family history, abortion history, and tumor size.

### miR-583

**Fig. 8:**
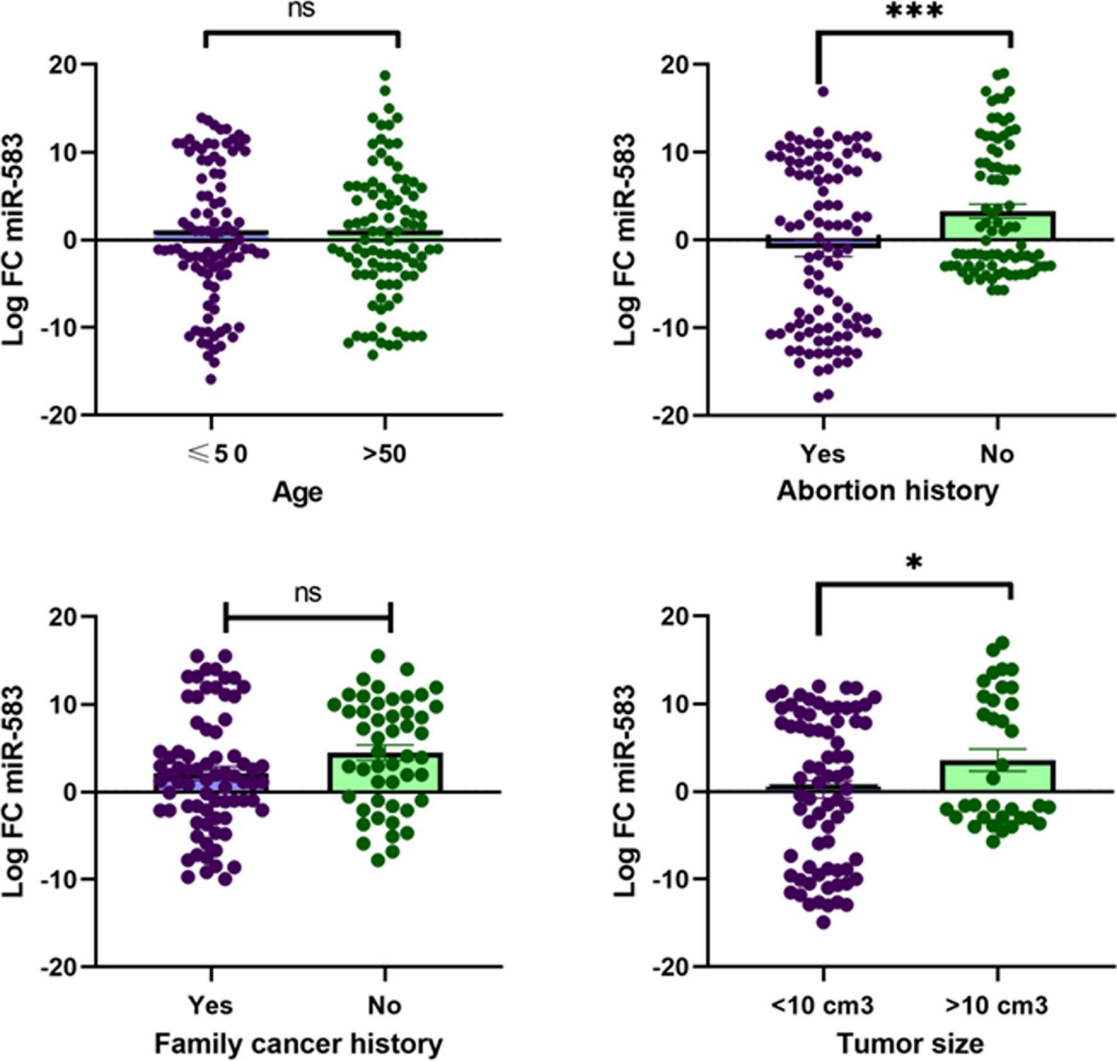
The correlation of miR‑583 expression status with age, abortion history, family cancer history, and tumor size. LFC miR‑583 in patients age ≥ 50 and < 50 (p = 0.974). LFC miR‑583 in patients with abortion history and without abortion history (***p < 0.0005). LFC miR-583 in patients with family cancer history and without family cancer history (p = 0.0521). LFC miR-583 in patients with tumor size < 10 cm^3^ and > 10 cm^3^ (*p = 0.049). In four figures, the vertical axis, center line, and error bars designate LFC (i.e., base 2 logarithm of FC), median, and interquartile range, respectively

Based on the results, there were no significant changes in the expression level of miR-583 in subgroups of age ≥ 50 and < 50 (p = 0.974) and in the subgroup of patients with and without family cancer history (Cancer family history means that a close relative has BC) (p = 0.0521). But there were significant differences in the expression level of miR-583 between patients with and without abortion history (***p < 0.0005) and patients with subgroups of tumor size < 10 cm^3^ and > 10 cm^3^ (*p = 0.049) **(**Fig. 8**)**.

### miR-877-5p

**Fig. 9:**
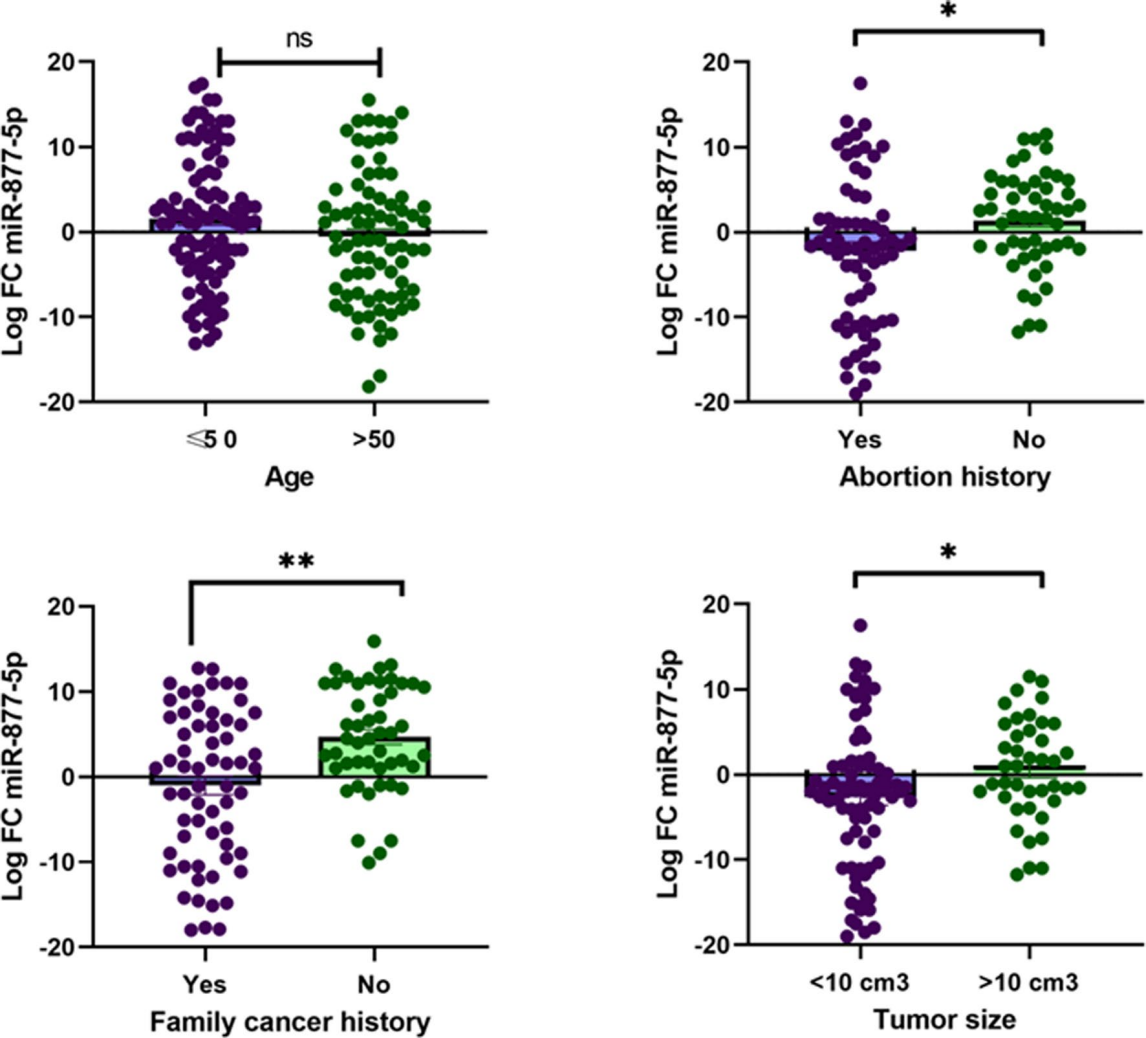
The correlation of miR‑877-5p expression status with age, abortion history, family cancer history, and tumor size. LFC miR‑877-5p in patients age ≥ 50 and < 50 (p = 0.767). LFC miR‑877-5p in patients with abortion history and without abortion history (*p = 0.0123). LFC miR-877-5p in patients with family cancer history and without family cancer history (**p = 0.0025). LFC miR-877-5p in patients with tumor size < 10 cm^3^ and > 10 cm^3^ (*p < 0.031). In four figures, the vertical axis, center line, and error bars designate LFC (i.e., base 2 logarithm of FC), median, and interquartile range, respectively

Based on the results, there were no significant changes in the expression level of miR-877-5p in subgroups of age ≥ 50 and < 50 (p = 0.767). Nevertheless, remarkably changes were seen in the expression level of the subgroup of patients with and without family cancer history (p = 0.0025), between patients with and without abortion history (p = 0.0123), and between patients with subgroups of tumor size < 10 cm^3^ and > 10 cm^3^ (p = 0.031) (Fig. 9**)**.

## Discussion

Breast cancer is a predominant cancer impacting females and resulting in high mortality [30]. MiRNAs have been determined to exert vital roles in the modulation of breast cancer. Previous studies have pinpointed several miRNAs in the regulation of breast cancer progression [31–33]. It has been reported that miR-513c and miR-3163 have a significant role in the development of BC [34]. Mir-335 is downregulated in breast cancer and is known as a tumor suppressor in BC [35]. Mir-30b-5p facilitates the Proliferation, migration, and invasion of breast tumors and acts as an oncomiR in breast cancer [36]. MiR-449b-5p as a tumor suppressor inhibits the growth and invasion of breast tumors by suppressing the Wnt/β-catenin signaling axis [37]. In this study, miR-583 and miR-877–5p were informed as novel breast cancer-related miRNAs that were downregulated in breast cancer tissues compared with adjacent normal tissues based on microarray and RT-qPCR results. Multiple studies’ analysis of the miRNA-mRNA network suggests that miR-583 and miR-877-5p, in association with certain proteins, contribute to the development of numerous malignancies. In 2020, Wu et al. declared that miR-877-5p inhibited gastric cancer growth and was known as a novel potential therapeutic target for gastric cancer [38]. In prostate cancer, miR-877-5p suppresses the malignant progression of cancer cells through miR-877-5p/SSFA2 axis [39]. Moreover, miR-877-5p as a tumor suppressor was detected in multiple available literatures published in 2018 [40–42] and 2020 [43] as a vital biomarker in patients with hepatocellular carcinoma.

Like miR-877-5p, the role of miR-583 as a biomarker has been proved in several studies. MiR-583 directly inhibits the proliferation and invasion of prostate cancer cells, providing a novel therapeutic target in prostate cancer [44]. Moreover, with the cooperation of other non-coding RNAs like circular RNAs, miR-583 hinders tumoral cells’ growth. Suppressing miR-583 through hsa_circ_0001955/miR-583/FGF21 axis promotes colorectal cancer [45]. These observations suggest miR-877-5p and miR-583 as tumor suppressors in cancer. To the best of our knowledge, miR-583 and miR-877-5p have not been reported in breast cancer.

Additionally, in this study, based on Enrichr and KEGG databases, the candidate targets of miR-583 and miR-877-5p might have an important role in endocytosis and pathways in cancer. Endocytosis is a potentially vital aspect in the regulation of tumor metastasis [46] and is confirmed in BC invasion and metastasis [47, 48]. However, pathways control various physiological functions and pathological events, in BC growth [49, 50]. These results suggest that the promising targets of miR-583 and miR-877-5p could be affected in the above-mentioned pathways to impact the occurrence and development of BC. In summary, in-silico and functional analysis results of the present study revealed that the downregulation of miR-583 and miR-877-5p promotes BC. However, the results of the RT-PCR elucidated the tumor suppressor role of miR-583 (p < 0.0001 and Log FC = − 1.35) and miR-877-5p (p < 0.0001 and Log FC = − 1.014) in BC tissues compared with adjacent non-tumor tissues. Furthermore, the demographic characteristics of our patients revealed that a significant difference was observed between the expression level of miR-583 and some demographic characteristics like abortion history and tumor size. Also, the expression level between miR-877-5p and family cancer history, abortion history, and tumor size were markedly different. The Kaplan-Meier result confirmed the prognostic value of miR-583 and miR-877-5p, and the ROC analysis result confirmed miR-583 (AUC = 0.69) and miR-877-5p (AUC = 0.64) great potential to be a valuable biomarker for BC.

## Conclusion

Taken together, downregulated miR-583 and miR-877-5p are potential molecular markers in BC. Our findings may suggest these miRNAs as promising diagnostic, prognostic, and therapeutic targets for BC, but further studies are needed to elucidate molecular mechanisms and validate the predicted findings using bioinformatics studies.

## Limitations

Some patients completed all the questions about the age, age of their first menstrual, age of menopause, family history, weight, number of children, underlying disease, medications used, age of breast cancer diagnosis, previous treatments, etc., themselves. Therefore, it’s probable that some patients didn’t answer the questions honestly and may have toned down the seriousness of their responses. The small number of surgical samples can be mentioned as another limitation.

## Data Availability

All datasets supporting the conclusions of this article are available in NCBI/bioproject: https://www.ncbi.nlm.nih.gov/bioproject/PRJNA116827, https://www.ncbi.nlm.nih.gov/bioproject/PRJNA182286, https://www.ncbi.nlm.nih.gov/bioproject/PRJNA195576, https://www.ncbi.nlm.nih.gov/bioproject/PRJNA248377, and https://www.ncbi.nlm.nih.gov/bioproject/PRJNA174219. Further inquiries can be directed to the corresponding author.

## Abbreviations

AUC: Curve and area under the curve
BC: Breast cancer
DEG: Differentially expressed genes
DEM: Differentially expressed miRNA
GO: Gene ontology
KEGG: Kyoto encyclopedia of genes and genomes
LFC: Logarithm fold change
MiRNA: MicroRNA
NcRNA: Noncoding RNA
One-way ANOVA: One-way analysis of variance
PCR: Polymerase chain reaction
ROC: Receiver operating characteristic
RT-qPCR: Reverse transcriptase-quantitative polymerase chain reaction
SD: Standard deviation
SEM: Standard error of the mean
WHO: World Health Organization
